# Phototheranostics of Cervical Neoplasms with Chlorin e6 Photosensitizer

**DOI:** 10.3390/cancers14010211

**Published:** 2022-01-02

**Authors:** Aida Gilyadova, Anton Ishchenko, Artem Shiryaev, Polina Alekseeva, Kanamat Efendiev, Radmila Karpova, Maxim Loshchenov, Victor Loschenov, Igor Reshetov

**Affiliations:** 1 University Clinical Hospital No. 1, Levshin Institute of Cluster Oncology, Sechenov First Moscow State Medical University, Ministry of Health of the Russian Federation, 119435 Moscow, Russia; aida-benyagueva@mail.ru (A.G.); artemdoc@mail.ru (A.S.); radmila.71@mail.ru (R.K.); reshetoviv@mail.ru (I.R.); 2 National Medical Research Center Treatment and Rehabilitation Center, Ministry of Health of the Russian Federation, 125367 Moscow, Russia; ra2001_2001@mail.ru; 3 Prokhorov General Physics Institute of the Russian Academy of Sciences, 119991 Moscow, Russia; alekseeva.polina2012@mail.ru (P.A.); maxvl@mail.ru (M.L.); loschenov@mail.ru (V.L.); 4 Department of Laser Micro-, Nano-, and Biotechnology, Institute of Engineering Physics for Biomedicine, National Research Nuclear University “MEPhI”, 115409 Moscow, Russia

**Keywords:** photodynamic therapy, photodiagnostics, phototheranostics, cervical cancer, cervical dysplasia, human papillomavirus, organ-preserving treatment

## Abstract

**Simple Summary:**

Neoplasms of the cervix are the most common types of oncological pathology. Photodynamic therapy with intravenous administration of the photosensitizer chlorin e6 shows high efficiency in the treatment of precancerous lesions of the cervix with complete eradication of the human papillomavirus. The treatment method can reduce deaths from cervical cancer and preserve fertility in patients. Spectral and video fluorescence diagnostics allows intraoperatively assessing the degree of photosensitizer accumulation and photobleaching and visualizing the boundaries of pathologically altered tissues.

**Abstract:**

(1) Purpose: Improving the treatment effectiveness of intraepithelial neoplasia of the cervix associated with human papillomavirus infection, based on the application of the method of photodynamic therapy with simultaneous laser excitation of fluorescence to clarify the boundaries of cervical neoplasms. (2) Methods: Examination and treatment of 52 patients aged 22 to 53 years with morphologically and cytologically confirmed mild to severe intraepithelial cervix neoplasia, preinvasive, micro-invasive, and squamous cell cervix carcinoma. All patients were carriers of human papillomavirus infection. The patients underwent photodynamic therapy with simultaneous laser excitation of fluorescence. The combined use of video and spectral fluorescence diagnostics for cervical neoplasms made it possible to control the photodynamic therapy process at all stages of the procedure. Evaluation of the photodynamic therapy of intraepithelial cervical neoplasms was carried out with colposcopic examination, cytological conclusion, and morphological verification of the biopsy material after the photodynamic therapy course. The success of human papillomavirus therapy was assessed based on the results of the polymerase chain reaction. (3) Results. The possibility of simultaneous spectral fluorescence diagnostics and photodynamic therapy using a laser source with a wavelength of 660 nm has been established, making it possible to assess the fluorescence index in real-time and control the photobleaching of photosensitizers in the irradiated area. The treatment of all 52 patients was successful after the first photodynamic therapy procedure. According to the PCR test of the discharge from the cervical canal, the previously identified HPV types were not observed in 48 patients. Previously identified HPV types were absent after repeated PDT in four patients (CIN III (*n* = 2), CIS (*n* = 2)). In 80.8% of patients, regression of the lesion was noted. (4) Conclusions. The high efficiency of photodynamic therapy with intravenous photosensitizer administration of chlorin e6 has been demonstrated both in relation to eradication therapy of human papillomavirus and in relation to the treatment of intraepithelial lesions of the cervix.

## 1. Introduction

Currently, cervical cancer is one of the most common types of oncological pathology [1]. According to the Global Cancer Observatory International Agency for Research on Cancer, 603,863 new cases of cervical cancer were registered in 2020. The absolute number of deaths caused by this disease was 341,680, which is 7.7% in the structure of mortality among women due to cancer [2]. The five-year survival rate of patients with cervical cancer in 2020 varied in different countries from 37 to 77% [3,4].

The etiological factor in most cases of squamous intraepithelial lesions of the cervix and squamous cell cervical cancer is the human papillomavirus (HPV) [3,4]. To date, more than 200 types of HPV have been described. Representatives of the Alpha genus, which mainly infect the mucous membranes of the anogenital tract, oral cavity, pharynx, and larynx [5,6].

Risk factors commonly associated with cervical cancer include younger age at sexual debut, multiple concurrent sexual partners, chronic heavy smoking, HIV infection, and persistent high-risk HPV infection.

A distinctive feature of cervical cancer is the ability to detect a previous condition, squamous intraepithelial damage to the cervix. At the same time, early intervention prevents the development of invasive forms of this pathology [7,8,9].

Squamous intraepithelial lesion of the cervix, characterized by squamous epithelial lesions (SIL), cervical intraepithelial neoplasia (CIN) in the form of atypical transformation of the squamous epithelium of the cervix without invasion into the stroma, is a precancerous condition [8,9,10]. Squamous intraepithelial cervical lesion is divided into the low-grade squamous intraepithelial lesion (LSIL) and high-grade squamous intraepithelial lesion (HSIL) [11].

The risk of developing cervical cancer in women with CIN is 20 times higher than in healthy women [12]. The average time for progression of CIN to cervical cancer is several years. Thus, the time interval for the diagnosis and treatment of CIN is sufficient [13,14].

Active screening among the female population for precancerous lesions and their treatment, if detected, especially in developing countries, provides very high chances of reducing mortality from cervical cancer and, given the prevalence of cervical pathology in young patients of reproductive age, the possibility of maintaining fertility [11]. 

To date, several methods have been proposed for diagnosing squamous intraepithelial lesions of the cervix. The main ones are extended colposcopic examination, cytological examination of scrapings of the ecto- and endocervix, molecular biological methods that allow identifying the HPV genotype with determining the degree of viral load, polymerase chain reaction (PCR), real-time PCR, and nucleic acid sequence-based amplification (NASBA) [10,11,12].

Optical diffuse reflectance spectroscopy is one technique that is believed to improve the efficiency and accuracy of screening and diagnosis of cervical lesions. When using this method, abnormal areas of the cervical epithelium illuminated by a low-power broadband light source provide backscatter spectra that differ from normal cervical tissues in the visible wavelength range. Such differences in the spectra detected by the optical sensor can be used to detect neoplastic epithelial lesions. In the optic tissue model, these parameters are associated with characteristics such as the size and density of the scatterers, the concentration of total hemoglobin, and oxygen saturation. These parameters can be used as optical markers for the assessment and classification of CIN centers. The disadvantages of the method as a screening and diagnostic tool are the variability of optical parameters and the lack of consistent values of threshold characteristics that can be used for diagnosis and choice of treatment strategy [13,14,15]. 

Fluorescence spectroscopy and visualization are based on the registration of absorption and emission of electromagnetic oscillations of the light range by a specific substance. Fluorescence spectroscopy for screening and diagnostics of cancer is based on the ability of this method to determine the molecular composition of tissue [14,15,16,17].

Ablation (thermal destruction of the affected tissue) and excision methods are used to treat CIN. Ablation methods include electro-, radio-, laser and cryodestruction. Excision methods of treatment include loop electrosurgical excision of the transformation zone, cold knife conization, and laser conization [18,19,20]. 

According to the methodological recommendations of the Russian Society of Specialists for the prevention and treatment of tumors of the reproductive system in young and/or planning pregnancy patients with a morphologically confirmed diagnosis of LSIL (coylocytosis, CIN I), expectant tactics with dynamic monitoring of the state of the cervix for 18–24 months, cytological control once every 6 months and HPV testing once every 12 months are preferable [21]. 

The above treatments are invasive. They lead to structural and anatomical changes in the cervix, which can negatively affect the reproductive capabilities of patients, the course and outcomes of pregnancy. Phototheranostics is a promising method for the diagnostics and treatment of cervical intraepithelial neoplasia and early invasive cervical cancer. It is a combined use of methods of spectral and video fluorescence diagnostics during photodynamic therapy (PDT) [19]. 

There are reports of the effectiveness of PDT in patients with CIN and HPV [18,20]. At the same time, the use of the method does not lead to adverse consequences for fertility [17,18,19,20]. The therapeutic effect of PDT is the formation of free radicals, such as singlet oxygen, which causes local photooxidation, damage, and destruction of specific cells [21]. This process is accompanied by the generation of excess reactive oxygen species to destroy tumor cells by inducing oxidative stress [19,20,21,22].

The results of studies [23,24] have demonstrated the high efficiency of PDT with chlorin e6 (Ce6) for the treatment of cervical neoplasms. Ce6 is a promising PS with significant diagnostic and therapeutic efficacy [25,26,27,28]. Ce6 is a second-generation PS with high efficiency and minimal dark phototoxicity [29]. The PS has intense absorption peaks at wavelengths of 402, 660–670 nm and intensive fluorescence in the range of 640 to 700 nm [30]. Ce6 accumulates mainly in blood cells and blood vessels. However, the Ce6 circulating through the blood vessels can easily diffuse into the tumor tissue due to the increased permeability and retention effect [31]. It is eliminated from the body or metabolized within the first 48 h after administration [32]. 

The study aimed to increase the effectiveness of treatment of intraepithelial cervical neoplasia associated with HPV infection, based on the phototheranostics method.

## 2. Materials and Methods

### 2.1. Patients

The study was conducted at I.M. Sechenov First Moscow State Medical University Clinical Hospital No. 1. It included 52 patients aged 22 to 53 years with morphologically confirmed intraepithelial neoplasia of the cervix. Each study was carried out in accordance with the Declaration of Helsinki by the World Medical Association. All studies had been approved by the Ethics Committee of First Sechenov State Medical University (L.L. Levshin Institute of Cluster Oncology). All patients signed informed consent to participate in the study. The distribution of the patients by the characteristics of lesions is shown in Table 1.

CIN I was diagnosed in 8 patients (15.4%), CIN II (19.2%) in 10, and CIN III (34.6%) in 18 women. Eight patients were diagnosed with preinvasive cervical cancer (CIS) (15.4%), 4 patients had cervical squamous cell carcinoma T1a1N0M0 (7.7%), and 4 patients had cervical squamous cell carcinoma T1b2N0M0, T2aN0M0 (7.7%).

Intraepithelial lesions of the cervix were verified using histological and cytological methods determined before PDT.

Before PDT, oncogenic HPV types were identified in all patients using PCR. HPV type 16 was detected in 20 patients, type 18 HPV in 16 women, type 6 in 6 patients, type 11 in 4 patients, type 35 in two patients, and type 56 in 4 patients.

The success of elimination therapy was assessed after PDT using the real-time PCR method. The treatment effectiveness of intraepithelial lesions of the cervix was studied based on cytological data using the liquid cytology method with Papanicolaou staining, as well as histologically after the end of the PDT course using targeted biopsy, which made it possible to assess the degree of therapeutic pathomorphosis. Patients with microinvasive cervical cancer underwent one procedure followed by conization. Two patients with stage T1b2N0M0 and T2aN0M0 squamous cell carcinoma of the cervix underwent a single PDT before extended panhysterectomy.

Cervical biopsies were fixed in 10% pH-buffered neutral formalin, subjected to standard histological automated processing on a Leica AutoStainerXL apparatus, followed by the preparation of paraffin blocks with tumor tissue samples and preparation of 5 μm sections. Slides were stained with hematoxylin-eosin. By light microscopy, the degree of the SIL was assessed following the WHO Classification of Tumors 5th edition Female Genital Tumor classification criteria.

For immunohistochemical studies, sections with a thickness of 4–5 µm were mounted on highly adhesive glasses, dried for 2–3 h at a temperature of 56–60 °C, and then for 18 h at a temperature of 37 °C. Staining was performed using an automatic immunostaining machine Ventana Benchmark XT kit (Ventana Medical Systems, Tucson, AZ, USA). Antibodies to p16 and Ki67 markers were used as molecular targets for HPV-positive and HPV-negative carcinomas. The results of immunophenotyping were evaluated following international recommendations. Expression of p16 was defined as positive if diffuse staining with strong nuclear and cytoplasmic expression was observed in the basal and parabasal layers of the stratified squamous epithelium of the cervix. Expression of Ki67 was defined as positive if diffuse staining with strong nuclear expression was observed in at least 2/3 of the layer of the stratified squamous non-keratinizing epithelium of the cervix. P16 is a surrogate marker for high-risk transcriptionally active HPV.

Before treatment the boundaries of the pathological areas of each patient were clarified using the combined spectral and video fluorescence diagnostics. 

### 2.2. Photosensitizer

In this study, we used commercially available photosensitizers (PS) Photolon^®^ (“Belmedpreparaty”, Belarus) and Photoran^®^ (Company “DEKO’’, Russia), the active component of which is the trisodium salt of chlorin e6 (Ce6). The maximum contrast of the Ce6 accumulation in the pathological tissue is observed 3–4 h after administration [32,33].

The calculated dose of Photolon^®^ or Photoran^®^ 0.8–1.2 mg/kg of body weight was dissolved in 150 mL of 0.9% sodium chloride solution. Each patient included in the study was infused with a solution prepared ex tempore 3 h before the start of treatment. PS was administered in a darkened room. Patients were instructed to strictly adhere to the light regime before and after PDT to avoid exposure to direct sunlight.

### 2.3. Video Fluorescence Diagnostics

**Figure 1:**
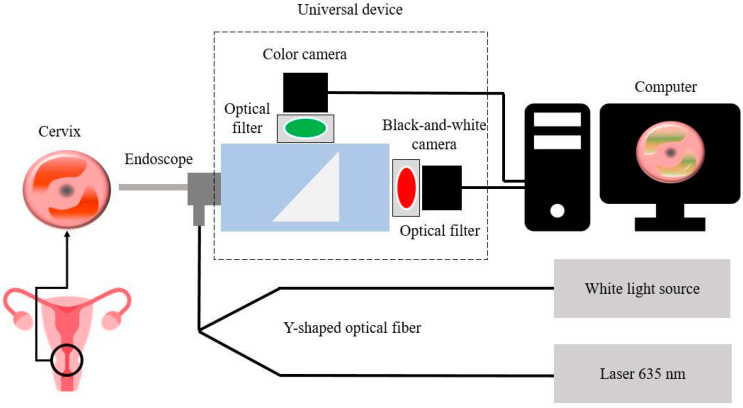
Scheme of video fluorescence diagnostics of areas of pathologically altered tissue.

For video fluorescence diagnostics, we used a two-channel fluorescent video system UFF-630/675-01-BIOSPEC (BioSpec, Moscow, Russia), consisting of a white light source; a laser radiation source with a wavelength of 635 nm; Y-shaped fiber cable for delivery of light to the surface of biological tissue; a universal device for recording backscattered and fluorescent radiation, equipped with a black-and-white digital charge-coupled device (CCD) camera and a digital color navigation camera; an endoscope (Figure 1).

A dichroic beam splitter is built into the universal device. Both radiation beams pass through optical filters. The color camera receives light after passing through a 625 nm short-pass filter, which transmits visible wavelengths up to 625 nm. The black-and-white camera receives radiation after passing through a 650 nm long-pass filter, which transmits wavelengths above 650 nm to pass through.

The two-channel video system allows receiving images in three modes: color, black-and-white (fluorescent), and combined. In the combined mode of the video system, the software turns on the synchronization of cameras and displays a color image in real-time. The fluorescent image is superimposed on top of the color image. Fluorescence is displayed in green, which allows visually determining the boundaries of the neoplasm.

**Figure 2:**
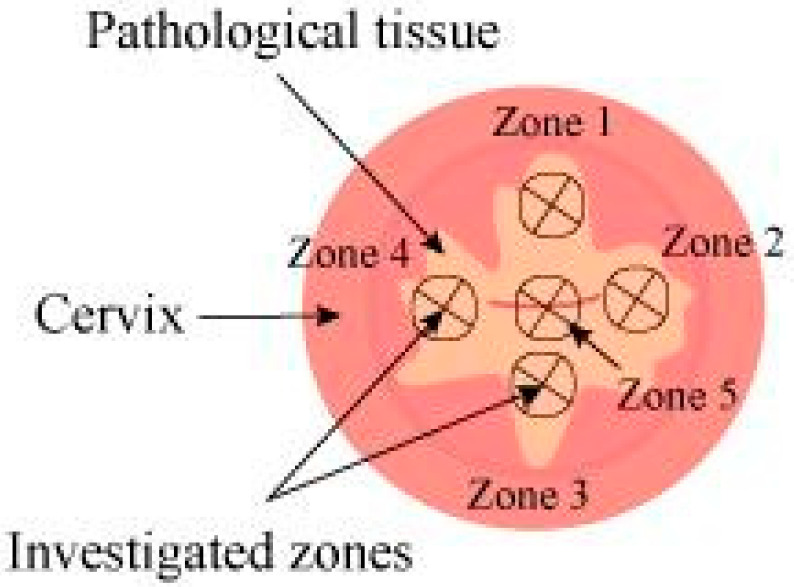
Scheme of video fluorescence diagnostics of areas of pathologically altered tissue.

In video fluorescence diagnostics, the degree of Ce6 accumulation in the mucous membranes of the cervix was quantified by the fluorescence index measured in the investigated zones 1–5, indicated in Figure 2.

Before the diagnostics of each patient, the fluorescence index was normalized. The areas with the lowest fluorescent signal (normal cervical mucosa) were assigned a fluorescence index value of 10 rel. units. Video fluorescence diagnostics allow identifying areas of the highest PS accumulation, which is typical for pathologically altered tissues (fluorescence index > 10 rel. units).

### 2.4. Spectral Fluorescence Diagnostics and Photodynamic Therapy

**Figure 3:**
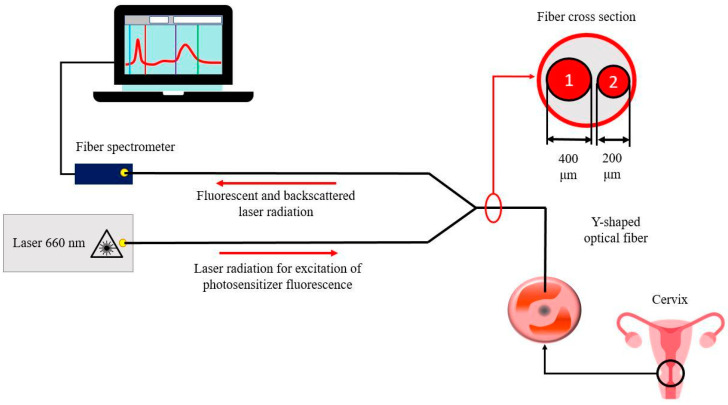
Scheme of spectral fluorescence diagnostics of areas of pathologically altered tissue.

For spectral fluorescence diagnostics, a fiber spectrometer LESA-01-BIOSPEC (BioSpec, Moscow, Russia) was used with excitation of PS fluorescence by a semiconductor laser LFT-02-BIOSPEC (BioSpec, Moscow, Russia) (λ = 660 nm). Fluorescence diagnostics was performed using a Y-shaped optical fiber equipped with two SMA-905 optical connectors (Figure 3).

Fluorescence spectra of normal and pathologically altered cervical tissue were recorded. Registration was carried out before and after PDT using a laser with a wavelength of 660 nm. In each case, the fluorescence index was calculated [34].

A laser with a wavelength of 660 nm is most convenient for simultaneous photodiagnostics (PD) and PDT. The advantage of this laser is the high power of the generated laser radiation (P = 2 W). PD and PDT using a laser source with a wavelength of 660 nm were carried out using a double Y-shaped optical fiber developed for this study. Thus, the PS photobleaching in the irradiated tissue during PDT was monitored in real-time by changing the fluorescence intensity.

Phototheranostics was carried out in 4 stages:

1. intravenous PS administered into the patient’s body;
2. conducting video fluorescence diagnostics;
3. carrying out spectral fluorescence diagnostics;
4. irradiation of pathological tissue accumulated PS with the light of the appropriate wavelength, PDT process.

Doses of light energy during PDT of the cervical mucosa were 100–250 J/cm^2^. The average power density for exposure to cervical tissue was 0.29 W/cm^2^, for the cervical canal is 0.25 W/cm^2^. An optical fiber with a cylindrical diffuser was used to irradiate the cervical canal. If the patient experienced pain during PDT, analgesics were used.

During spectral and video fluorescence diagnostics, the investigated areas were periodically washed from blood or blood secretions.

## 3. Results

**Figure 4:**
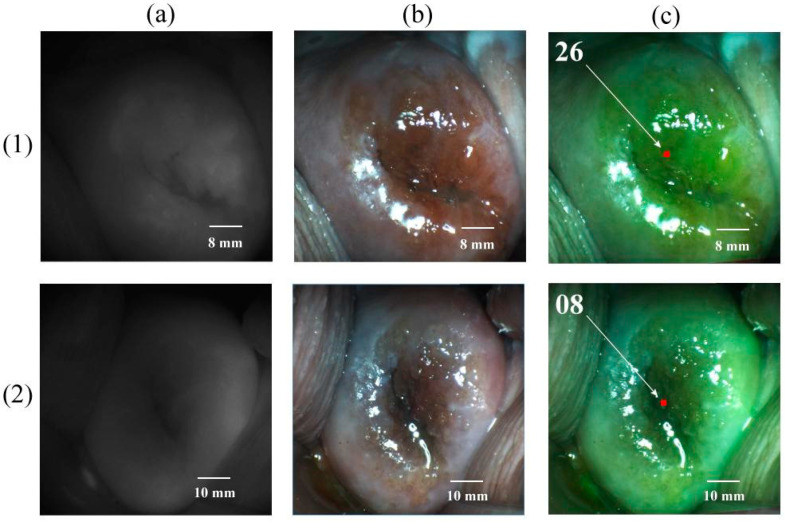
Images of cervical tissue from a patient with CIN III associated with HPV 6 and 16. (**1**) Before PDT. (**2**) After PDT. (**a**) Black-and-white mode. (**b**) Color mode. (**c**) Combined mode (in the upper left corner of the image is the fluorescence index in rel. units).

All patients included in the study tolerated intravenous Ce6 well. Mild skin phototoxicity was observed within 24 h after PS administration in 6 patients. Images of the cervical tissue before and after PDT were obtained in all patients. Figure 4 shows images of the cervical tissue of patients with CIN III associated with HPV 6 and 16.

Before PDT, the fluorescence index of the pathologically altered tissue was 26 rel. units. After PDT, the fluorescence index decreased to 08 rel. units, which indicated a decrease in the fluorescence intensity of pathologically altered tissue after PDT due to PS photobleaching to values that correspond to the characteristics of normal tissue (fluorescence index ≤10 rel. units).

**Figure 5:**
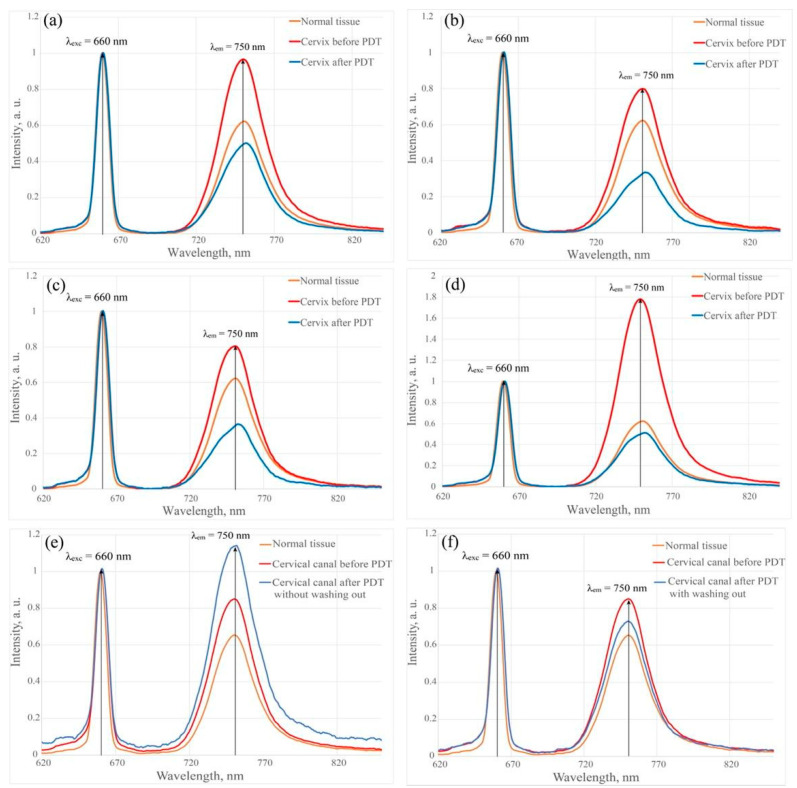
Results of spectral fluorescence diagnostics of cervical tissues of a patient with CIN III. The fluorescence spectra normalized to the laser line of the healthy and pathologically altered cervical tissue of the patient with CIN III before and after PDT using a laser with a wavelength of 660 nm in the investigated zones. (**a**) Zone 1. (**b**) Zone 2. (**c**) Zone 3. (**d**) Zone 4. (**e**,**f**) Zone 5.

Figure 5 shows the results of spectral fluorescence diagnostics of cervical tissues of a patient with CIN III before and after PDT in the investigated zones 1–4 using a laser with a wavelength of 660 nm.

The fluorescence intensity in zones 1–3 after PS administration increased by almost 30%. The intensity in Zone 4 increased by 65% relative to normal tissue. A significant increase in fluorescence intensity in one of the investigated zones may indicate the presence of a higher degree of damage, in which the ability of the tissue to accumulate PS in high concentrations increases.

The fluorescence intensity decreased in all investigated zones of the cervix by 60% after PDT due to PS photobleaching. In the tissues of the cervix, the penetration depth of laser radiation for wavelengths of 675 and 780 nm is 2.40 ± 0.22 and 2.61 ± 0.25 mm, respectively [35]. The use of a single source of laser radiation for PD and PDT underlies the method of phototheranostics.

**Figure 6:**
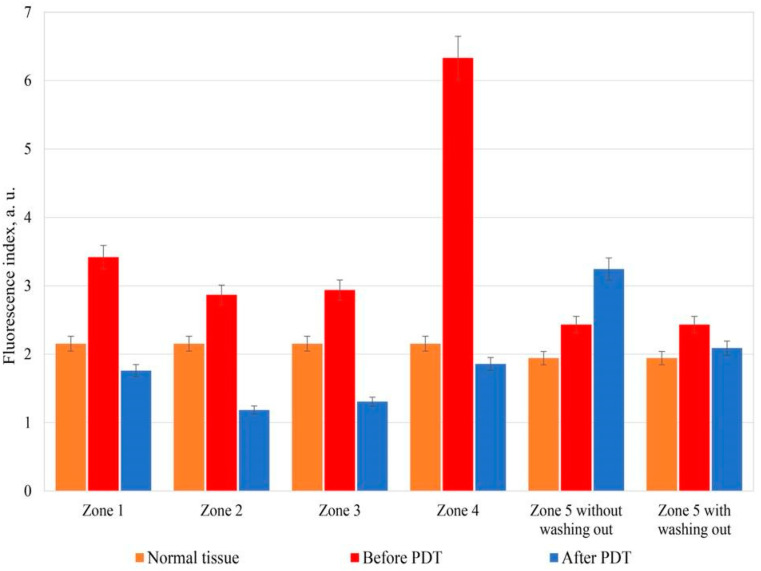
Fluorescence indices of healthy and pathologically altered tissue of the patient cervix with CIN III before and after PDT in the investigated zones 1–5.

Figure 6 shows the fluorescence indices of the healthy and pathological tissue of the patient cervix with CIN III before and after PDT in the investigated zones 1–5.

In the investigated zones 1–3 and 5, the fluorescence index after PS administration increased by about 30%. In Zone 4 the fluorescence index increased by 66% relative to healthy tissue.

After PDT, the fluorescence index decreased under the influence of therapeutic radiation by an average of 60% relative to the results before PDT in the investigated zones 1–4. The fluorescence index in Zone 5 increased by 20% after PDT before the cervix washing out from the PS relative to the values before PDT. However, after the cervix washing out with 0.9% aqueous sodium chloride solution and repeated spectral fluorescence diagnostics, Ce6 photobleaching of 14% was reliably detected (Figure 6). During spectral and video fluorescence diagnostics, the cervical canal was periodically washed out from blood or blood secretions due to the high content of Ce6 in the secretions. The secretions, an additional absorber, can significantly reduce the depth of the photodynamic effect and worsen the results of the reliability of fluorescence diagnostics, which can lead to insufficient light exposure of sensitized tissues of the cervix or cervical canal, can cause a relapse of the disease.

**Figure 7:**
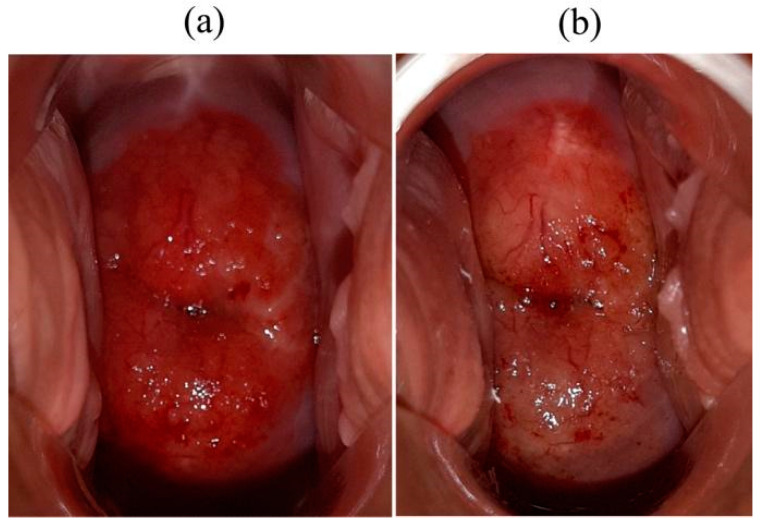
Cervix of the patient with CIN. (**a**) Before PDT. (**b**) Immediately after the end of PDT.

Edema and hyperemia were observed in the irradiated areas of the pathologically altered tissue (Figure 7).

Necrotic formations in the investigated zones of the cervix were detected 2–3 days after PDT. Chin W.W. et al. (2006), based on the analysis of the results of colposcopy and fluorescent images, revealed a correlation between the necrotic formation in the irradiated area of the pathologically altered tissue and PS photobleaching observed immediately after PDT [24].

In most cases, the fluorescence index of pathological tissue after PDT decreased by two or more times compared to 10 rel. un., which is a sign of the high efficiency of the treatment method.

PDT effectiveness of CIN was assessed based on colposcopy, the cytological conclusion of the Papanicolaou test, the liquid cytology, and morphological verification of the biopsy material after the end of the PDT.

Successful treatment and complete regression of the pathologically altered tissues were noted in all patients with CIN I, CIN II, CIN III, and CIS. It was confirmed by the results of cytological and histological studies three months after PDT.

The distribution of patients depending on the number of PDT procedures, which made it possible to achieve a clinical effect, is shown in Table 2. According to the PCR test of the discharge from the cervical canal, the previously identified HPV types were not observed in 48 patients. Previously identified HPV types were absent after repeated PDT in 4 patients (CIN III (*n* = 2), CIS (*n* = 2)). The absolute majority (80.8%) of women showed regression of the lesion after one PDT.

The distribution of patients with different characteristics of cervical lesions, depending on the number of PDT procedures, which made it possible to achieve a clinical effect, is shown in Table 3.

All patients with CIN I (*n* = 8) and CIN II (*n* = 10) required one PDT procedure to achieve a complete response. In 12 of 18 (66.7%) patients with CIN III after the first PDT procedure, the conclusion of the cytological and histological examination corresponded to the norm (NILM). In the remaining 6 patients (33.3%) with CIN III after the first PDT procedure, the conclusion of the cytological study corresponded to mild dysplasia (CIN I). These patients underwent the second PDT procedure. Morphological verification of the diagnosis after the repeated PDT procedure in these patients indicated the absence of pathologically altered tissues, which corresponds to the complete regression of the disease.

In 4 of 8 (50%) patients with CIS, complete disease regression was noted after a single PDT procedure. In 4 patients, after the first PDT, CIS regressed to LSIL/CIN I, and a complete response was obtained after the second PDT.

In 4 patients (100%) with microinvasive squamous cervical cancer T1a1N0M0 after a single PDT, conization of the cervix was performed. After the histological study, the changes corresponded to the mild squamous intraepithelial lesion of the cervix, which, in combination with conization of the cervix, corresponded to a complete response to treatment.

A single PDT before extended panhysterectomy was performed in 4 patients with squamous cell cervical cancer T1b2N0M0 and T2aN0M0. In the histological report of the postoperative material, 2 patients with stage T1b2N0M0 showed moderate dysplasia of the cervix, and 2 patients (50.0%) with stage T2aN0M0 showed therapeutic pathomorphosis of a grade 2 tumor according to the scale of assessment of therapeutic tumor pathomorphism according to G.A. Lavnikova [35].

According to the data of extended colposcopy with 3.5% solution of acetic acid performed before PDT, abnormal signs were observed in 2 patients with CIN II, 18 patients with CIN III, and 4 patients with CIS. In 3 women with invasive cervical cancer, grade II was determined in the form of dense acetone epithelium, rough mosaic, and rough puncture; in 4 patients with preinvasive cervical cancer and 2 patients with invasive cancer, a ridge symptom was determined.

In 3 patients with invasive cervical cancer, colposcopic signs of invasion with atypical vessels against the background of a rough mosaic were revealed. In 8 patients with CIN I and 8 women with CIN I-II, mild signs of damage to the cervix in the form of a delicate mosaic, puncture, and thin aceto-white epithelium were noted.

The results of colposcopy carried out after the first PDT showed the colposcopic picture was normal in all patients. There was no aceto-white epithelium when tested with 3.5% acetic acid solution. Iodine negative zones were not detected in Schiller’s test.

**Figure 8:**
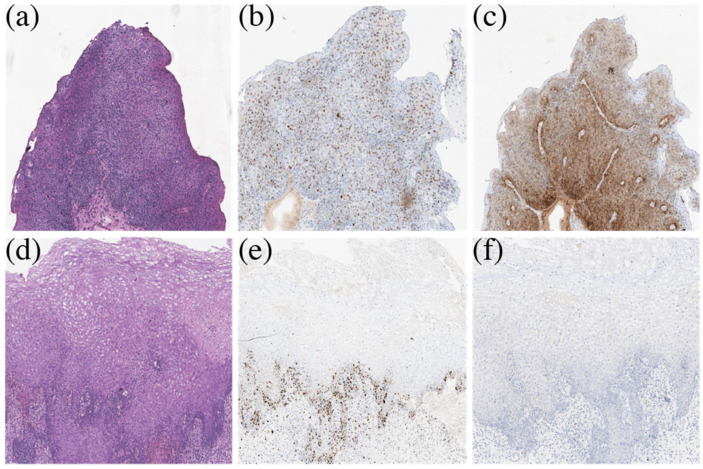
Results of histological examination of cervical biopsies of a 41-year-old patient with HSIL. (**a**–**c**) Lesion before PDT. (**d**–**f**) Lesion after PDT.

Figure 8 shows the results of histological examination of cervical biopsies of a patient with high-grade squamous intraepithelial lesion before PDT (a) and 1 month after PDT (d). Therapeutic pathomorphosis of the lesion, which was qualified as a complete response to the therapy, due to the absence of the morphological picture of CIN, was identified. The figure shows samples of immunohistochemical staining for the Ki67 marker, respectively, before (b) and after PDT (e). The figure demonstrates p16 immunohistochemical staining samples before (c) and after PDT (f). After treatment, a significant decrease in the color intensity is observed, which indicates a significant decrease in the expression of markers Ki67 and p16 in the epithelium of the cervix.

The results obtained indicate the high efficiency of PDT with intravenous Ce6 administration for the treatment of intraepithelial lesions of the cervix with a selective effect on pathologically altered tissue.

## 4. Discussion

Early diagnostics and treatment of precancerous cervical diseases is the most effective measure for the prevention of cervical cancer. PDT is an alternative organ-preserving therapeutic approach to the treatment of CIN and CIS [1,7]. Phototheranostics combine diagnostics and therapy in one procedure. Our data are consistent with the results of studies by other researchers.

The possibility of treatment of CIN and CIS using PDT methods has been demonstrated in several studies. Thus, 24 patients with CIN underwent PDT using a 1% solution of the PS Photofrin, of which 13 had signs of CIN I, 7 had CIN II, and 4 had CIN III. The study used different doses of light with a power range of 100–140 J/cm^2^. Successful treatment and complete regression of neoplastic lesions were achieved in 15 (68%) patients [36]. Other authors report that after PDT CIN II, III, and CIS using Photofrin, complete remission was observed in 20 out of 22 cases (91%) at an energy density of 240 J/cm^2^. In this case, the cervix and cervical canal was exposed to laser irradiation [37].

It has been shown that the use of Ce6 in PDT promotes deeper necrosis since the absorption wavelength of radiation when using this approach is in the “biological transparency window” from 650 to 950 nm. It has been demonstrated that the action mechanism of Ce6 depends on the PS accumulation in the vasculature, not in tumor tissue. Its effectiveness is mainly explained by the destruction of the vascular system of neoplasms with PDT. Ce6 has an intense absorption band between 640 and 680 nm with a maximum at about 660 nm and intensive fluorescence in the range of 640 to 700 nm [37].

The results of the relatively low PDT efficiency with 5-aminolevulinic acid (5-ALA) in the CIN treatment are presented in several reports [17,38,39,40,41]. A randomized clinical trial compared the efficacy of topical application of 3% 5-ALA gel (13 patients) and placebo (13 patients) during CIN therapy. As a result, there were no statistically significant intergroup differences in treatment outcomes [17].

Barnett A. A. et al. (2003) showed that 5-ALA-induced PDT with argon laser in 7 women with high CIN was not efficient [40].

The low efficiency of PDT for CIN, reported by some researchers, may be associated with insufficient processing of the cervical canal because the topical PS application was used in the presented works.

The best results were achieved with intravenous 5-ALA in the treatment of CIN. A group of researchers reported the PDT effectiveness in 31 patients with CIN II and CIN III [41]. In 100% of cases, complete regression of CIN was revealed 12 months after the end of treatment. It also reported the successful treatment of 105 women with CIN I and CIN III using PDT with intravenously administered Photofrin II. In 94 of 105 (90%) women, complete regression of CIN was observed 3 months after laser irradiation of the vagina and cervical canal with PDT [42].

The results of our study demonstrated the high PDT efficiency with Ce6 in patients with CIN I, II, III, and CIS. In one of the works, the authors reported on the high efficiency of PDT with Ce6 for CIN I and CIN II at doses of light energy of 100–200 J/cm^2^. The study included 112 patients with CIN I and CIN II. In 104 (92.8%) women, complete regression of CIN was detected. Complete eradication of HPV infection was confirmed by PCR test in 47 (53.4%) of 88 patients infected with HPV 16, 18, 31, 33 3 months after PDT [43].

Thus, the high action selectivity on the affected cervical tissues, a low risk of developing adverse reactions and complications, a short period of systemic photosensitivity, and high therapeutic efficacy distinguish PDT with Ce6 from traditional methods of CIN I, II, III, and CIS treatment, which are accompanied by local destruction of the injured tissues. PDT with Ce6 is an efficient, relatively safe, and minimally invasive method for the CIN and CIS treatment that allows achieving HPV infection eradication.

## 5. Conclusions

The phototheranostics method is a promising effective and safe therapeutic and diagnostic approach for precancerous cervix neoplasm. The results indicate that Ce6 is characterized by high photodynamic activity. Video fluorescence diagnostics allows the most accurate identification of the boundaries of the pathologically cervix tissue. Parallel application of spectral-fluorescent diagnostics makes it possible to estimate Ce6 distribution in the investigated tissues.

The study demonstrated the possibility of simultaneous spectral fluorescence diagnostics and PDT using a laser source with a wavelength of 660 nm, which made it possible to assess the fluorescence index in real-time and control the PS photobleaching in the irradiated area. The combined use of video and spectral fluorescence diagnostic methods for detecting neoplasms of the cervix allows controlling the PDT process at all stages of the procedure. The results show that phototheranostics is an effective and minimally invasive method for treatment cervix neoplasm, which contributes to the elimination of HPV infection. The clinical effectiveness of the presented method is demonstrated by a significant improvement in the colposcopic picture. In patients with histologically confirmed neoplasia and cervical cancer after PDT, abnormal signs of lesion and invasion were not determined.

Thus, PDT contributes to the successful treatment of cervical tissue pathologies. The effectiveness of the method is ensured by the selectivity of the effect on pathologically altered tissues. PDT does not result in damage to normal surrounding tissues, rough scarring, and stenosis of the cervical canal. Thus, the method allows preserving anatomical and functional characteristics of the cervix. It is a significant criterion for the preservation of fertility in patients, which confirms the advantage of PDT for the intraepithelial cervical neoplasia compared with alternative methods of treatment, such as ablative, excisional, and conization of the cervix.

## Figures and Tables

**Table 1:** Distribution of patients according to the characteristics of the lesions of the cervix (*n* = 52).

Characteristics of Pathologies	Number of Patients	
	Abs.	%
CIN I	8	15.4
CIN II	10	19.2
CIN III	18	34.6
CIS	8	15.4
Microinvasive squamous cervical cancer	4	7.7
Squamous cervical cancer	4	7.7

**Table 2:** Distribution of patients by the number of PDT procedures (*n* = 52).

NPLR *	NP **	
	Abs.	%
1	42	80.8
2	10	19.2

* NPRL—the number of procedures after which there was lesion regression; ** NP—number of patients.

**Table 3:** Distribution of patients by characteristics of cervical lesions (*n* = 52).

Characteristics of Pathologies	NP1 *		NP2 **	
	Abs.	%	Abs.	%
CIN I (*n* = 8)	8	100.0	-	-
CIN II (*n* = 10)	10	100.0	-	-
CIN III (*n* = 18)	12	66.7	6	33.3
CIS (*n* = 8)	4	50.0	4	50.0
Microinvasive squamous cervical cancer (*n* = 4)	4	100.0	-	-
Squamous cervical cancer (*n* = 4)	4	100.0	-	-

* NP1—the number of patients for whom the effect was observed after one PDT procedure; ** NP2—the number of patients for whom the effect was observed after two PDT procedures.

## Data Availability

The data presented in this study are available on request from the corresponding author.
